# Effects of Immersive Technology–Based Education for Undergraduate Nursing Students: Systematic Review and Meta-Analysis Using the Grading of Recommendations, Assessment, Development, and Evaluation (GRADE) Approach

**DOI:** 10.2196/57566

**Published:** 2024-07-24

**Authors:** Subin Park, Hui Ju Shin, Hyoeun Kwak, Hyun Joo Lee

**Affiliations:** 1 College of Nursing Yonsei University Seoul Republic of Korea; 2 Severance Hospital Yonsei University Health System Seoul Republic of Korea; 3 Mo-Im Kim Nursing Research Institute College of Nursing Seoul Republic of Korea; 4 Yonsei Evidence-Based Nursing Centre of Korea: A Joanna Briggs Institute-Affiliated Group Seoul Republic of Korea

**Keywords:** nursing education, nursing students, immersive technology, systematic review, meta-analysis, virtual reality, augmented reality, extended reality, simulation-based learning, medical education

## Abstract

**Background:**

The adoption of immersive technology in simulation-based nursing education has grown significantly, offering a solution to resource limitations and enabling safe access to clinical environments. Despite its advantages, there are still diverse reports regarding the effectiveness of immersive technology. It is crucial to verify the effectiveness of immersive technology in nursing education to inform future educational programs.

**Objective:**

This systematic review aimed to identify the contents of immersive technology–based education for undergraduate nursing students and evaluate the effectiveness of immersive technology compared to traditional teaching methods.

**Methods:**

A literature search was performed using 4 databases: PubMed, CINAHL, Embase, and Web of Science; the latest search was completed on January 19, 2023. The inclusion criteria were as follows: participants were undergraduate nursing students; studies were published in Korean or English; designs included randomized controlled trials (RCTs) or nonrandomized studies; and interventions involved virtual reality (VR), augmented reality (AR), mixed reality, or extended reality. Quality assessment was conducted using Cochrane Risk-of-Bias Tool version 2 for RCTs and the Risk-of-Bias Assessment Tool for Nonrandomized Studies. The main outcomes of the included studies were classified according to the New World Kirkpatrick Model (NWKM), ranging from level 1 (reaction) to level 4 (results). Meta-analysis was conducted using RevMan 5.4 software, and subgroup analysis was conducted due to heterogeneity of the results of the meta-analysis. The Grading of Recommendations, Assessment, Development, and Evaluation approach was adopted for assessing certainty and synthesizing results of the relevant literature.

**Results:**

A total of 23 studies were included, with participant numbers ranging from 33 to 289. Of these, 19 (82.6%) studies adopted VR to simulate various nursing scenarios, including disaster training, resuscitation, health assessments, and home health care; 4 (17.4%) studies used AR technologies; and 15 (65.2%) studies involved virtual patients in their scenarios. Based on the NWKM, the main outcome variables were classified as level 1 (usability and satisfaction), level 2 (knowledge, motivation, confidence, performance, attitude, and self-efficacy), and level 3 (clinical reasoning); level 4 outcomes were not found in the selected studies. Results of the subgroup analysis showed that immersive technology–based nursing education is more effective than traditional education in knowledge attainment (standard mean difference [SMD]=0.59, 95% CI 0.28-0.90, *P*<.001, I^2^=49%). Additionally, there were significant difference differences between the experimental and control group in confidence (SMD=0.70, 95% CI 0.05-1.35, *P*=.03, I^2^=82%) and self-efficacy (SMD=0.86, 95% CI 0.42-1.30, *P*<.001, I^2^=63%).

**Conclusions:**

These findings support the effectiveness of immersive technology–based education for undergraduate nursing students, despite heterogeneity in methods and interventions. We suggest that long-term cohort studies be conducted to evaluate the effects of immersive technology–based nursing education on NWKM level 4.

## Introduction

### Background

Immersive technology is widely recognized to improve learning in nursing education [1,2]. The idea of immersive technology emerged 6 decades ago with the human-computer prototype known as the “Man-Machine Graphical Communication System” [3]. Immersive technology is derived from the reality-virtuality continuum concept and encompasses virtual reality (VR), augmented reality (AR), and mixed reality (MR) [2]. “Extended reality” (XR), an umbrella term that includes all 3 technologies, is recognized as a type of immersive technology [4]. VR refers to technology that enables users to immerse themselves in virtual worlds and interact with them, while AR involves adding virtual elements to the real environment to merge reality with virtuality. MR integrates VR and AR to provide an experience where the real and virtual environments interact, and the degree of immersion is related to the stimulated senses, interactions, and similarities between reality and virtuality [5]. According to Cipresso et al [5], VR is classified into 3 technology types based on the degree of immersion: nonimmersive technology involves using desktop computers to reproduce images of virtual worlds, semi-immersive technology uses perspective projection to provide stereo images of 3D scenes viewed on a monitor, and immersive technology represents the highest level of technical immersion, providing users with a sense of presence in virtual environments [5].

A significant goal of nursing education is the transfer of theoretical knowledge to clinical practice [6]. However, limited clinical resources impact students’ opportunities to gain hands-on experience with patients, and the lack of hands-on experience in clinical practice may pose a risk of patient safety when the students face challenges in a real-world clinical environment in the future as health professionals [7,8]. Therefore, nursing educators should provide students with sufficient alternative clinical experiences [7]. To ensure the quality of nursing education in clinical practice, educators have incorporated various educational strategies, including simulations [9]. Various clinical simulation methods have been developed [10]. In the nursing field, there is a growing interest in using immersive technology as an effective educational tool for simulation-based programs to enhance students’ knowledge and skill acquisition [4,11].

Immersive technology reduces the limitations of the resources required for 2D simulation–based learning [12]. Education programs adapted to immersive technology enable students to access clinical practice with ease and develop their skills within a secure setting, minimizing risks to patient safety [12,13]. According to Foronda et al [14], 98% of participants expressed a preference for incorporating virtual learning environments. There have been efforts to further the leverage of immersive technology, especially with the increased significance of remote classes due to the outbreak of COVID-19 [15,16]. Additionally, because immersive technology improves the interaction between students and instructors by facilitating discussions, it is frequently used in simulation-based learning [17]. Student-instructor interaction helps derive successful outcomes when properly supported with high-fidelity simulations [18].

### The New World Kirkpatrick Model

The Kirkpatrick Model, developed by Donald L Kirkpatrick in 1959 and expanded in 1967, is a widely used framework for evaluating the effectiveness of educational programs. This model categorizes program outcomes into 4 levels [19]. Level 1 encompasses participant reactions, assessing how favorable, engaging, and relevant they find the training to be in relation to their jobs. Level 2 includes the learning outcomes; at this level, the focus is on the knowledge, skills, attitude, confidence, and commitment acquired by learners because of training. Level 3 evaluation is related to changes in the participants’ behavior based on the simulation experience. Critical behavior must have a few key actions that are performed consistently by the primary group to bring about the targeted outcome. Level 4 is the final outcome evaluation, which indicates the actual changes in the output or results due to the training. In 2010, the New World Kirkpatrick Model (NWKM) emerged, presenting a framework with 4 levels of evaluation that is more effectively applicable to the current changing circumstances [19]. It modifies the direction of the result levels in reverse, in the order of levels 4-1 [19,20]. The NWKM proposes planning eventual program outcomes in the planning stage. Some outcomes have been added to each level of evaluation, and parts of the definitions have been revised. Both quantitative and qualitative methods can be used to evaluate each level, and this model has been widely used to evaluate the outcomes of education programs in the nursing field [21-23].

### Study Objective

Although there are evident advantages to using immersive technology in nursing education, there are claims suggesting that it may not be notably effective compared to traditional teaching methods, such as didactic lectures, use of audiovisual materials, and students’ practice following the instructor’s demonstration [9,24-28]. In addition, many studies have verified the effectiveness of VR methods in nursing education [9,29,30]; however, there remains a shortage of studies that comprehensively assess the effectiveness of immersive technology encompassing all concepts of VR, AR, MR, and XR. Therefore, this study aimed to identify the contents of immersive technology–based education programs for undergraduate nursing students and evaluate the effectiveness of the interventions.

## Methods

### Reporting Guidelines

This study adhered to the Preferred Reporting Items for Systematic Review and Meta-Analysis (PRISMA) guidelines [31]. The study protocol was preregistered in the International Prospective Register of Systematic Reviews (PROSPERO; registration number CRD42023400085).

### Eligibility Criteria

Eligibility criteria were established based on the Population, Intervention, Comparison, and Outcome (PICO) framework. The target population was undergraduate nursing students. The search for studies included randomized controlled trials (RCTs) and nonrandomized studies that used VR, AR, XR, and MR technologies. Regarding the immersion of VR technology, the search specifically focused on studies that used head-mounted devices (HMDs), including glasses, goggles, and helmets—the most immersive and extensively used visual devices in VR technology [5]. Other immersive technologies, such as AR, XR, and MR, encompassed all devices such as smartphones and smart glasses [5]. The outcome variables were not restricted to the search and were categorized according to the NWKM [19]. Theses and dissertations, along with studies not published in either English or Korean, and those designed as pilot studies or case studies were excluded.

### Search Strategy

A thorough search was conducted across 4 databases: PubMed, CINAHL, Embase, and Web of Science. Search terms were selected judiciously, adhering to the principles of the Medical Subject Headings (MeSH), with specific terms customized for each database (Multimedia Appendix 1). Additionally, consultation with a librarian at the medical library informed and refined the search strategy. The search was conducted on January 19, 2023. Search records were imported into the reference management tools EndNote (Clarivate) and Covidence, a specialized program for systematic reviews. Following the application of Covidence’s artificial intelligence (AI) function to automatically identify and remove duplicate studies, manual confirmation was performed. After eliminating duplicates, the remaining studies underwent eligibility screening by 2 independent reviewers (authors SP and HJS) according to predefined inclusion and exclusion criteria. In cases of discrepancies between the 2 reviewers, a third reviewer (author HK) was consulted to reach a consensus. Finally, all researchers agreed on the final literature to be included in the analysis. The initial screening process involved the assessment of titles and abstracts for relevance. Subsequently, full-text screening was performed, and the rationale for exclusion was documented in the PRISMA flowchart.

### Quality Assessment

The quality assessment of all RCTs was performed based on Cochrane Risk-of-Bias Tool version 2 (RoB 2) [32]. RoB 2 consists of 5 key domains that evaluate potential biases in study design and conduct. These domains are related to the randomization process, intended intervention, absence of outcome data, and selective reporting of results. Individual domains were assessed for their potential influence on the validity of the findings. The quality assessment of nonrandomized studies was based on the Risk-of-Bias Assessment Tool for Nonrandomized Studies (RoBANS) [33]. The domains encompass issues such as bias stemming from participant selection, confounding variables, measurement, blinding, incomplete outcome data, and selective reporting of results. Two reviewers (SP and HJS) independently assessed the quality of each of the 23 included studies. For the assessment results of 22 (95.7%) studies, there was agreement between the 2 reviewers; however, 1 (4.3%) study on which agreement was not reached between the 2 reviewers was reassessed by a third reviewer (HK).

### Data Extraction

An exclusive data extraction template was used to collect pertinent details from each study: author, publication year, country, research design, participants, and sample size. Regarding immersive technology interventions, the extracted items included the type of technology, content characteristics, the length and duration of the intervention, facilitator details, the presence of prebriefing and debriefing sessions, scenarios, and the VR content development company. The data extraction template was filled in independently by 2 reviewers (SP and HJS). For disagreements, the third reviewer (HK) reevaluated the papers and facilitated consensus among the researchers based on clear evidence. Finally, a fourth reviewer (author HJL) reviewed and confirmed the overall content. For outcome variables, the extracted information included the measurement timing, evaluated variables, measurement tools, and classification based on the NWKM, and mean (SD) values were extracted for subsequent meta-analysis. Outcome variables were classified into the 4 levels of the NWKM: level 4 (whether the organization exists to perform, deliver, or contribute to its customers or society at a high level), level 3 (critical behaviors, required drivers, and on-the-job learning), level 2 (confidence and commitment highlighted to close the gap between learning and behavior, along with intended knowledge, skills, and attitude), and level 1 (measures such as satisfaction and usability to assess the extent to which participants perceived the education as positive, engaging, and relevant) [19].

### Statistical Analysis

RevMan 5.4 software was used to synthesize the data [34]. The overall effect size was calculated using the SMD, along with the 95% CI, as the studies used different measurements for each outcome. For pre- and posttests, if changes in the measurement variables were not reported, the correlation between the 2 covariances was assumed to be 0.5 [35], and the changes and measurement variances of the variables were reported accordingly. The effect size was classified into small (0.2), medium (0.5), large (0.8), or very large (1.2) based on Cohen’s guidelines. To analyze the overall effect, Z-statistics were applied at a significance level of *P*<.05. Heterogeneity was estimated using the Higgins I^2^ statistic, which provided insight into the degree of variation among the included studies. Heterogeneity can be interpreted as nonobserved (0%), low (0%-25%), moderate (25%-50%), or high (>50%) [36]. The random effects model was used for data analysis due to the presence of heterogeneity in the mediation process across various scenarios and measurement variables [37]. The results of the meta-analysis were presented as forest plots.

### Assessment of Certainty of Evidence

The Grading of Recommendations, Assessment, Development, and Evaluation (GRADE) approach was used to assess the quality of evidence. This involved evaluating the study design, risk of bias, inconsistency, indirectness, imprecision, and other relevant factors. Based on these evaluations, the quality of evidence was rated on a 4-point scale: high, moderate, low, or very low [38].

## Results

### Study Selection

**Figure 1:**
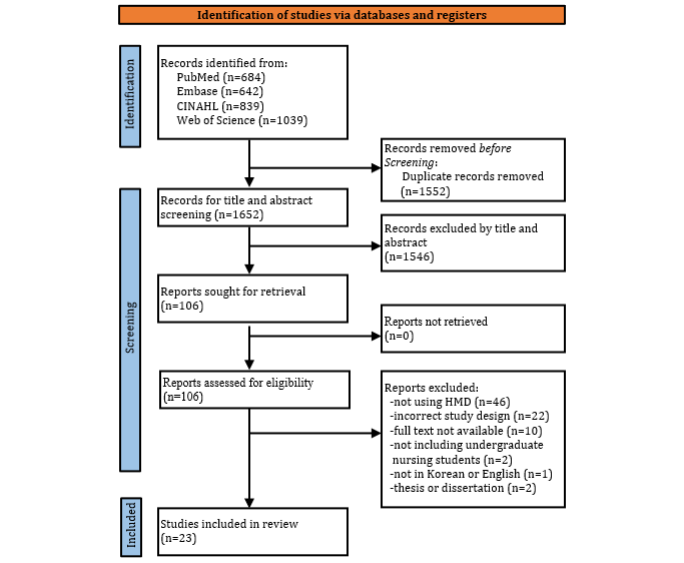
PRISMA flow diagram of the study selection. HMD: head-mounted device; PRISMA: Preferred Reporting Items for Systematic Review and Meta-Analysis.

Figure 1 shows the process of study selection based on the PRISMA 2020 flow diagram. A total of 3204 studies were identified by searching the 4 databases. The AI function of Covidence automatically removed 1534 (47.9%) duplicates. In addition, 18 (0.6%) studies were manually identified as duplicates. Of the remaining 1652 (51.6%) papers, 1546 (93.6%) were excluded after a thorough review of their titles and abstracts. The full text of the remaining 106 (6.4%) papers was screened, and 83 (78.3%) papers were excluded for the following reasons: not related to immersive technology (eg, VR not using an HMD; n=46, 55.4%), incorrect study design (n=22, 26.5%), unavailable full text (n=10, 12%), not including undergraduate nursing students (n=2, 2.4%), not in Korean or English (n=1, 1.2%), and theses or dissertations (n=2, 2.4%). Finally, 23 studies (n=22, 95.7%, in English and n=1, 4.2%, in Korean) were selected.

### Study Characteristics

Table 1 presents the study characteristics. The studies included in this research were conducted in 8 countries: China [39], Finland [40], Norway [41,42], South Korea [43-48], Spain [49,50], Taiwan [51-55], Turkey [56,57], and the United States [58-61]. All studies were published within the past 5 years, except for 1 (4.3%) study [60] published in 2018; notably, there was a significant increase in the number of studies published in 2021 (n=10, 43.5%). The number of participants ranged from a minimum of 33 to a maximum of 289. Of the 23 studies, 21 (91.3%) were conducted with nursing students as the primary participants, whereas the remaining 2 (8.7%) specifically targeted both medical and nursing students. In addition, 6 (26.1%) studies were designed as RCTs, while 17 (73.9%) were nonrandomized studies. Furthermore, 4 (17.4%) studies [40,44,51,54] used AR, whereas the remaining 19 (82.6%) used VR in their educational programs. The experimental group participated in VR or AR simulation programs, whereas the control group received traditional education or no intervention.

**Table 1:** General characteristics of included studies (N=23).

Author	Country	Type of immersive technology	Study design	Participants	Total sample size, N
Shujuan et al [39]	China	VR^a^	RCT^b^	Second-year nursing students of a tertiary program, registered for the disaster nursing course	101
Havola et al [40]	Finland	VR	Nonrandomized study	Graduating nursing students from a single university	40
Berg and Steinsbekk [41]	Norway	VR	RCT	First-year medical/nursing students	289
Berg and Steinsbekk [42]	Norway	VR	RCT	First-year medical/nursing students	289
Ahn and Lee [43]	South Korea	VR	Nonrandomized study	Third-year nursing students	84
Jung and Park [44]	South Korea	VR	Nonrandomized study	Second-, third-, and fourth-year nursing students	60
Lee and Han [45]	South Korea	VR	Nonrandomized study	Fourth-year nursing students	60
Yang and Oh [46]	South Korea	VR	Nonrandomized study	Prelicensure nursing students	83
Yu et al [47]	South Korea	VR	Nonrandomized study	Senior nursing students	50
Yu and Yang [48]	South Korea	VR	Nonrandomized study	Third- and fourth-year nursing students	50
Rodríguez-Abad et al [49]	Spain	AR	Nonrandomized study	Second-year nursing students	137
Mayor Silva et al [50]	Spain	VR	RCT	First-year students from the Faculty of Nursing	100
Chang et al [51]	Taiwan	VR	Nonrandomized study	Nursing students from a single nursing college	64
Chao et al [52]	Taiwan	VR	RCT	Nursing students	45
Chen et al [53]	Taiwan	VR	Nonrandomized study	Third-year nursing students	79
Chen and Liou [54]	Taiwan	AR^c^	RCT	Fourth-year nursing students	95
Wu et al [55]	Taiwan	VR	Nonrandomized study	Third-year nursing students from a single university, 9 pediatric classes	105
Kurt and Öztürk [56]	Turkey	AR	Nonrandomized study	First-year nursing students	122
Sen et al [57]	Turkey	VR	Nonrandomized study	Second-year nursing students taking the operating room nursing course	40
Dang et al [58]	United States	VR	Nonrandomized study	Prelicensure, baccalaureate nursing students in their first medical-surgical course	160
Herbert et al [59]	United States	AR	Nonrandomized study	Second-semester junior nursing students	33
Smith et al [60]	United States	VR	Nonrandomized study	Senior baccalaureate nursing students, recruited from 4 different Midwest university campuses	172
Smith et al [61]	United States	VR	Nonrandomized study	Senior nursing students in the final semester of a baccalaureate nursing program	121

^a^VR: virtual reality.

^b^RCT: randomized controlled trial.

^c^AR: augmented reality.

### Intervention Characteristics

#### Scenario Features

The intervention scenarios covered a wide range of nursing situations, including disaster training [5,48,61], resuscitation [40,46,54], nursing education [44,45,47,50-52,55,57,58], health assessments [53], and home health care nursing [43]. The virtual locations for the intervention scenarios varied, including settings such as the patient’s home [43] and clinical environments, such as general wards [52,55], the emergency room [60], intensive care units (ICUs) [40,45], neonatal intensive care units (NICUs) [46,47], delivery rooms [51], angiography rooms [44], operating rooms [57], and isolation units [48]. Of the 23 studies, 15 (65.2%) [39-48,53-55,60,61] mentioned that they featured virtual patients. Of these, 7 (46.7%) studies [39,41-43,54,55,58] allowed for interaction between the virtual patient and the learner. This interaction involved assessing the virtual patient’s health status through the airway, breathing, circulation, disability, and exposure (ABCDE) approach [41,42] or providing nursing interventions following the assessment of the patient’s condition [39,43,54,55]. However, in 1 (14.3%) study [58], it was unclear whether there was any interaction between the virtual patient and the learner.

#### Implementation of Immersive Technology

Various devices were used to operate the immersive technology. Of 19 (82.6%) studies that used VR technology, 11 (57.9%) [39,40,44-46,50-52,55,57,58] operated the scenario using only HMDs (ie, VR goggles, glasses, headsets, and helmets) with embedded controllers, while 9 (47.4%) studies [41-43,46-48,53,60,61] used haptic devices and motion trackers for controlling and tracking their motions in a virtual environment. The remaining 4 (17.4%) studies [49,54,56,59] used AR technology operated with smartphones or tablets so that they could augment fidelity via lenses and screens.

#### Administration of Immersive Technology–Based Education

Regarding learning methods, 1 (4.3%) study [53] used immersive technology in a 15-week classroom lecture, and 1 (4.3%) study [42] used a team-based approach. The other studies conducted interventions independently. The length of the scenarios ranged from 8 to 110 minutes, with some studies not specifying a time limit or providing explicit information regarding the scenario length. In addition, 10 (43.5%) studies [43-48,50,55,58,60] included both prebriefing and debriefing sessions, 7 (30.4%) studies [39,40,52-54,59,61] included only prebriefing sessions, 2 (8.7%) studies [49,56] included only debriefing sessions, and 1 (4.3%) study [51] included neither prebriefing nor debriefing. Furthermore, 10 (43.5%) studies [43,44,50-54,56,58,59] did not describe the role of the instructor. In the remaining studies, the instructor provided minimal intervention, offering only technical support when learners engaged with immersive technology.

### Outcome Variables

The outcome variables of each study were classified using the NWKM [19], as shown in Table S1 in Multimedia Appendix 2. The outcomes included satisfaction [41,42,44,45,47,48,51,52,59-61], usability [41,42,58], a sense of realism [46,58], anxiety [46], knowledge [39,41-48,50-60], confidence [39,43,52,54,57,61], self-efficacy [43,45,47,48,61], performance [39,43,45,46,48-50,53,54,56,57,60,61], attitude [44,51], motivation [44,46,49,51], critical thinking [51], and clinical reasoning [40,46]. The outcomes were categorized into NWKM levels 1-3; no outcome variable corresponded to level 4.

### Risk of Bias

**Figure 2:**
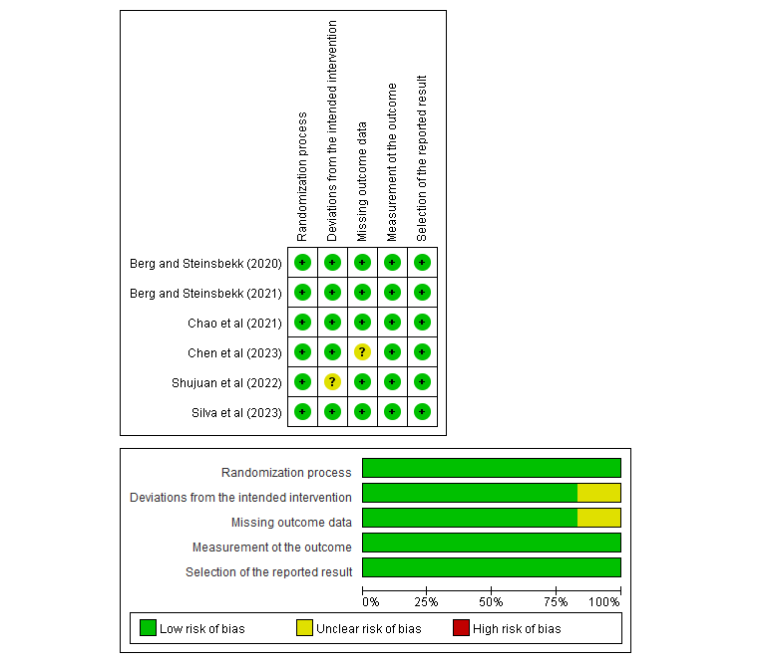
Risk-of-bias summary of RCTs. RCT: randomized controlled trial.

**Figure 3:**
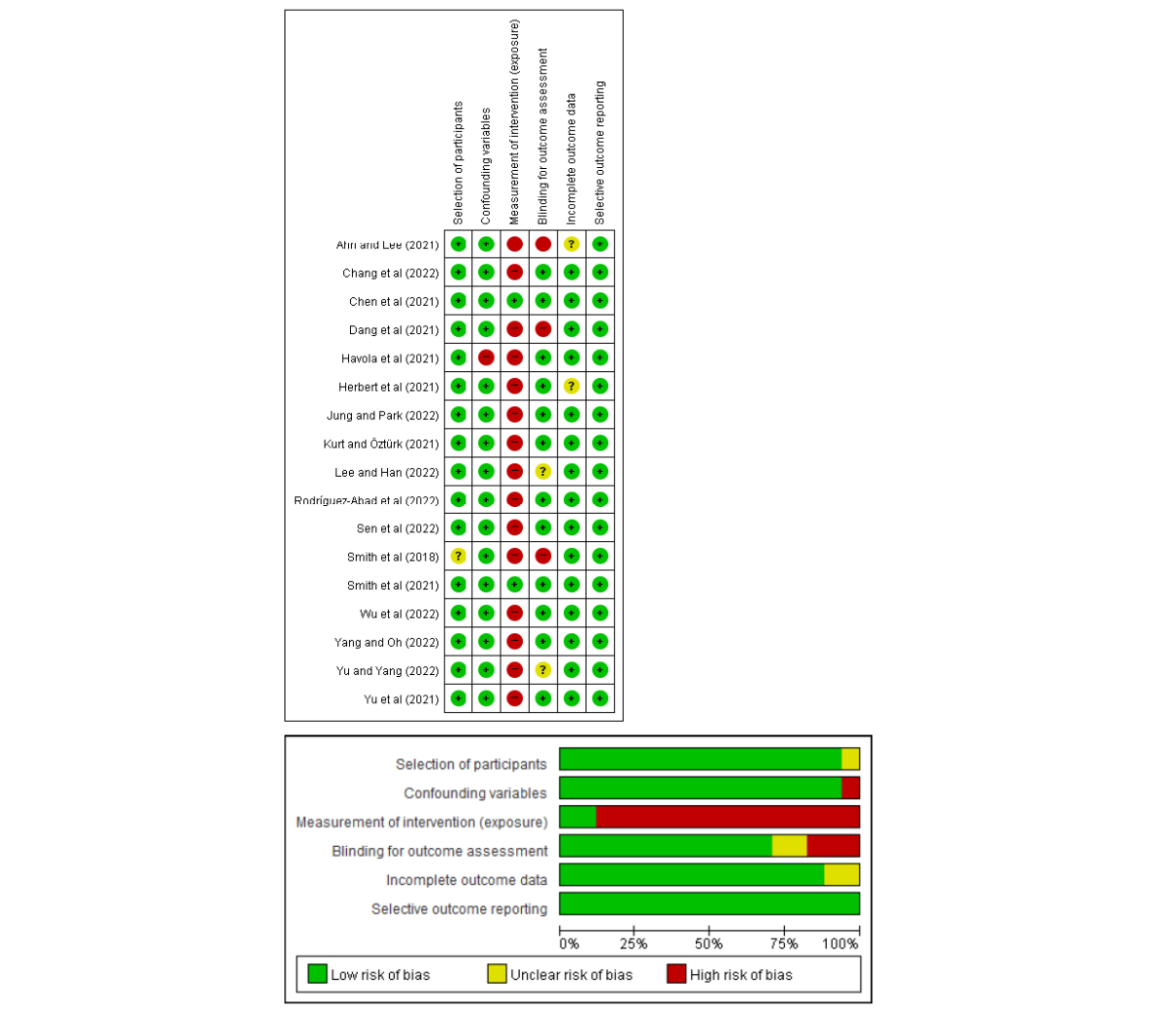
Risk-of-bias summary of nonrandomized studies.

Figures 2 and 3 show the results of risk-of-bias assessment. RoB 2 was used to appraise 6 RCTs [39,41,42,50,51,53], of which 4 (66.7%) [41,42,50,52] confirmed all components to have low risk, while for the other 2 (33.3%) studies, the risk of bias was unclear for the items of missing outcome data [54] and deviation from the intended intervention [39]. RoBANS was used to assess the quality of 17 (73.9%) nonrandomized studies [40,43-49,51,53,55-61], of which 15 (88.2%) [40,43-49,51,55-60] had a high risk of bias in the measurement of the intervention. They used self-reported methods to assess outcome variables. In the incomplete outcome data category, 15 (88.2%) studies reported a low dropout rate, making them suitable for classification under a low risk of bias, while 2 (11.8%) studies [43,59] were categorized as having an unclear risk of bias. One nonrandomized study [51] was reevaluated to resolve discrepancies in quality assessment, ultimately reaching a consensus.

### Meta-Analysis Findings

#### Effects of Immersive Technology–Based Education on Students’ Knowledge Attainment

**Figure 4:**
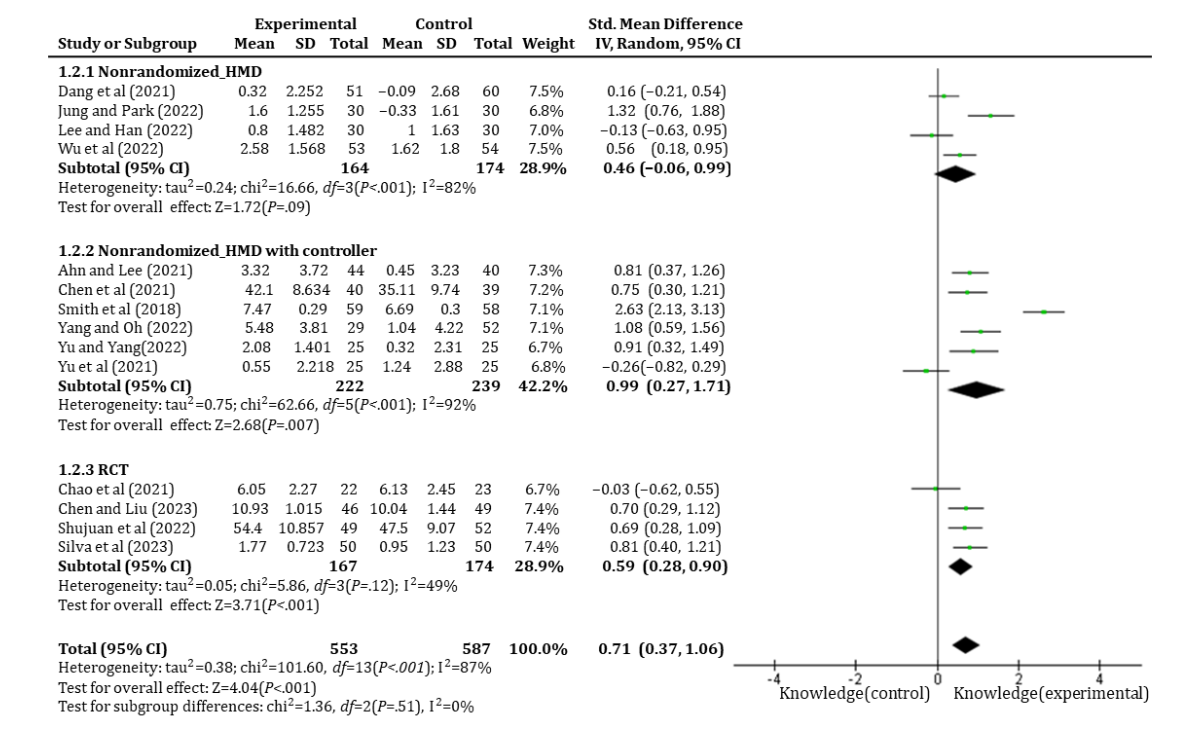
Forest plot of immersive technology–based education on students’ knowledge acquisition. HMD: head-mounted device.

**Figure 5:**
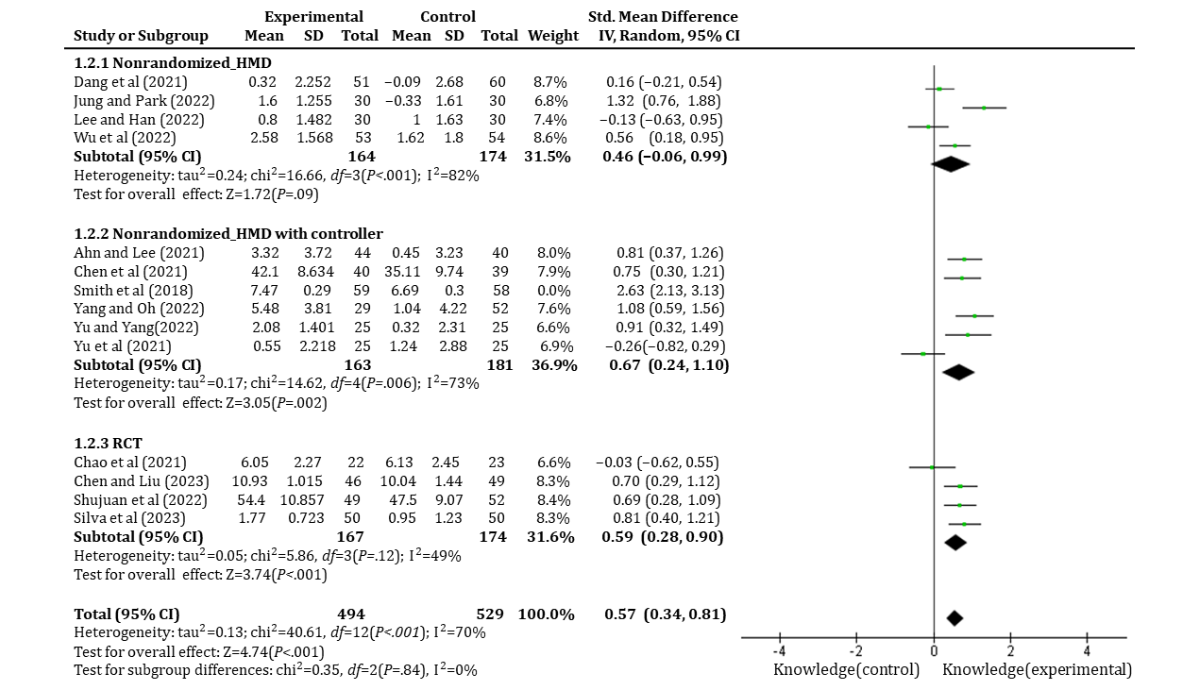
Sensitivity analysis results of in nonrandomized studies using HMDs with controllers. HMD: head-mounted device.

Of the 23 studies, 14 (60.9%) studies [39,43-48,50,52-55,58,60] involving 553 students were analyzed to evaluate the effects of immersive technology–based education on students’ knowledge attainment. Of the 14 studies, 10 (71.4%) [39,40,43,45-48,54,55,58] used nonrandomized design and the other 4 (28.6%) [39,50,52,53] used RCTs. The findings revealed that the experimental group significantly enhanced students’ knowledge (SMD=0.71, 95% CI 0.37-1.06, *P*<.001); however, significant heterogeneity (I^2^=87%, *P*<.001) was observed between the 14 (60.9%) studies. To address this heterogeneity, subgroup analysis was conducted based on study design to distinguish between RCTs and nonrandomized studies. In the RCTs, a reduction in heterogeneity was observed (SMD=0.59, 95% CI 0.28-0.90, *P*<.001, I^2^=49%, *P*=.12). Further subgroup analysis was performed on the nonrandomized studies based on the type of equipment used. The results showed that studies using only HMDs did not significantly enhanced students’ knowledge (SMD=0.46, 95% CI –0.06 to 0.99, *P*=.09, I^2^=82%, *P*<.001), while studies using both HMDs and controllers significantly enhanced students’ knowledge (SMD=0.99, 95% CI 0.27-1.71, *P*=.007, I^2^=92%, *P*<.001), as shown in Figure 4. Sensitivity tests were additionally carried out for nonrandomized studies using HMDs and controllers, except 1 (4.3%) study [60] published in 2018. The results are shown in Figure 5, and I^2^ reduced to 73%. However, the test for subgroup differences indicated no statistically significant subgroup effect (*P*=.84), implying that the study design does not modify the effect of knowledge attainment. Nevertheless, a fewer number of trials provided data for the RCT subgroup compared to the nonrandomized study subgroup, potentially limiting the ability of the analysis to detect subgroup disparities (Figure 5).

#### Effects of Immersive Technology–Based Education on Students’ Confidence

**Figure 6:**
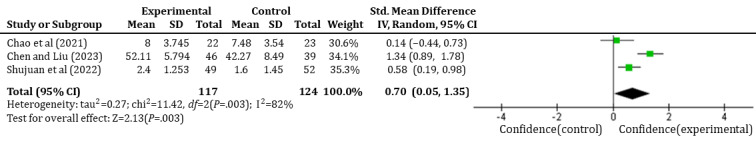
Forest plot of immersive technology–based education on students’ confidence.

Of the 23 studies, 3 (13%) studies [39,52,53] involving 117 students were analyzed to evaluate the effects of immersive technology–based education on students’ confidence. All 3 (13%) studies were designed as RCTs and used VR. The findings revealed that compared to control conditions, the interventions for the experimental group significantly enhanced students’ confidence with a medium effect size (SMD=0.70, 95% CI 0.05-1.35, *P*=.03). The heterogeneity test showed a high level of heterogeneity across the studies (I^2^=82%, *P*<.001), as shown in Figure 6.

#### Effects of Immersive Technology–Based Education on Students’ Self-Efficacy

**Figure 7:**
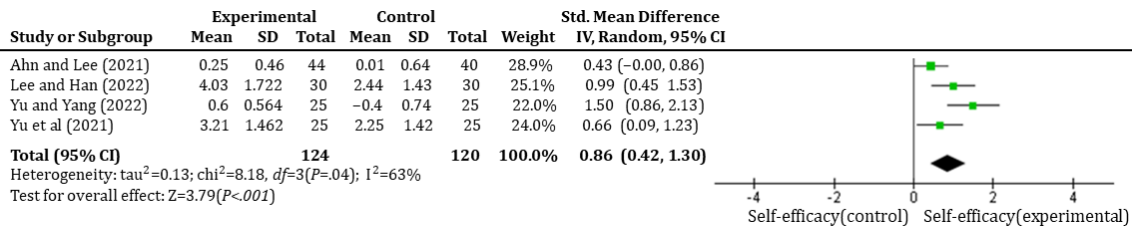
Forest plot of immersive technology–based education on students' self-efficacy.

Of the 23 studies, 4 (17.4%) studies [43,45,47,48] involving 120 students were analyzed to evaluate the effects of immersive technology–based education on students’ self-efficacy. Only nonrandomized studies were included, with immersive technology using VR. The findings revealed that compared to control conditions, the interventions significantly enhanced students’ self-efficacy with a large effect size (SMD=0.86, 95% CI 0.42-1.31, *P*<.001). The heterogeneity test showed a high level of heterogeneity across the studies (I^2^=63%, *P*=.04), as shown in Figure 7.

### Quality of the Evidence

The results of GRADE assessment are shown in Table 2. The evidence for knowledge outcomes in RCTs was rated as high, while that for confidence outcomes in RCTs was rated as low. Conversely, the evidence for knowledge outcomes in the nonrandomized study design subgroup, which included the use of HMDs or additional controllers, was rated as very low and low, respectively. Similarly, the evidence for self-efficacy was also rated as low.

**Table 2:** Summary of findings using GRADE^a^.

Study design	Outcomes	Participants, N; studies (N=23), n (%)	SMD^b^ (95% CI)	Heterogeneity	Quality of evidence
RCT^c^	Knowledge	341; 4 (17.4)	0.59 (0.29 to 1.27)	I^2^=49%, *P*=.12	High (imprecision, large magnitude of effect)
RCT	Confidence	241; 3 (13.0)	0.70 (0.05 to 1.35)	I^2^=82%, *P*<.001	Low (inconsistency, imprecision, large magnitude of effect)
Nonrandomized	Knowledge (HMD^d^)	338; 4 (17.4)	0.46 (–0.06 to 0.99)	I^2^=82%, *P*<.001	Very low (risk of bias, inconsistency, imprecision)
Nonrandomized	Knowledge (HMD^d^ with controller)	344; 5 (21.7)	0.67 (0.24 to 1.10)	I^2^=73%, *P*=.006	Low (risk of bias, inconsistency, imprecision, large magnitude of effect)
Nonrandomized	Self-efficacy	244; 4 (17.4)	0.86 (0.42 to 1.31)	I^2^=63%, *P*=.04	Low (risk of bias, inconsistency, imprecision, large magnitude of effect)

^a^GRADE: Grading of Recommendations, Assessment, Development, and Evaluation.

^b^SMD: standard mean difference.

^c^RCT: randomized controlled trial.

^d^HMD: head-mounted device.

## Discussion

### Principal Findings

This systematic review identified the effectiveness of immersive technology in nursing education and assessed the quality of evidence according to the GRADE approach. Of the 23 studies selected, 19 used VR and 4 used AR, with 22 of the 23 studies published within the past 5 years. This underscores the contemporary relevance of immersive technology–based education in current teaching and learning methods. In addition, 18 studies implemented scenario-based interventions, which covered a diverse range of health care scenarios from clinical settings to home health care nursing, while 15 studies incorporated virtual patients; notably, in 7 of these studies, learners interacted with the virtual patients, allowing them to practice nursing care similar to that provided to actual patients but within a safe environment. Scenario-based learning, which encompasses diverse patient populations in various settings, has become essential in nursing education. In this context, immersive technology that implements computer-generated virtual environments has proven to be an effective approach in enhancing the effects of scenario-based education [2,9]. Furthermore, 4 studies indicated the educational effectiveness of observation in simulation settings. Observation is an advanced learning method in nursing practice, which can be advantageous if it is planned appropriately with pedagogical theories and resources in simulation-based education [62]. Observers can acquire new knowledge through objective perspectives. Immersive technology has the potential to develop a method of observing in the field of nursing education, which is presently restricted by limitations of resources.

Advanced technological equipment for implementing immersive technologies continues to be developed. Immersive technologies were facilitated by the use of equipment such as controller-embedded HMDs, haptic devices, and motion trackers in 19 studies, reflecting the latest trends in technology used in education. In addition, the 4 studies using AR technologies also used smartphones and tablets already owned by learners, enhancing accessibility and cost-effectiveness by capturing the real environment and overlaying digital images. The continuous development of various advanced devices necessitates that educators select equipment that effectively supports the achievement of learning objectives.

The outcome variables of the studies comprised 3 dimensions aligned with the NWKM, and the findings align with the current challenge in assessing the long-term effects of nursing education on professional nursing practice. To overcome this challenge, it is necessary to first plan how to assess the long-term achievement of educational programs prior to undertaking the programs. To facilitate the evaluation of the long-term outcomes at level 4 of the NWKM, it is imperative to undertake follow-up research, which can provide the ascertained efficacy of immersive technology–based nursing education [63].

According to the results of the meta-analysis in this study, compared with traditional learning methods, immersive technology–based nursing education is effective in improving undergraduate nursing students’ knowledge attainment, confidence, and self-efficacy. Additionally, heterogeneity was observed among the studies measuring the effects on knowledge acquisition. A subgroup analysis based on research design and the equipment used moderately reduced the heterogeneity, and significant effects on knowledge acquisition were reported in RCTs. Nonrandomized studies using HMDs showed no significant effect on knowledge acquisition, while those that combined HMDs with controllers showed positive effects. The level of evidence through GRADE for knowledge acquisition in RCTs was high based on the study design, a low risk of bias, moderate heterogeneity, and a large effect size, while that in nonrandomized studies (HMDs with controllers) was downgraded due to the study design, a high risk of bias, high heterogeneity, and imprecision but upgraded for a large effect size, resulting in an overall rating of low. Therefore, the results should be interpreted carefully as future research might yield divergent findings. Moderate-to-high heterogeneity across studies underscores the need for the development of standardized guidelines to design immersive technology–based education and gold-standard tools to measure the efficacy of educational programs.

### Limitations and Future Research

This study has several limitations. The major limitation was the large heterogeneity between studies, which requires careful interpretation of the research findings. Various types of software and equipment were used for interventions, and the results from each study varied owing to differences in technical functions. To ensure the quality of the studies, we included only those published in peer-reviewed journals. However, published research often emphasizes only significant results, posing a risk of reporting bias. Furthermore, the majority of the identified studies were published within the past 5 years, and additional studies may have been published since the completion of the review in January 2023.

Despite these limitations, this review comprehensively analyzed the characteristics of immersive technology–based education, providing valuable insights for educators and researchers aiming to implement such technologies in their teaching. We recommend the use of interactive virtual patients with scenario-based learning and the selection of devices that enhance interaction, such as HMDs or haptic devices, while considering learning objectives and practicality, including cost-effectiveness. Additionally, RCTs show that groups receiving immersive technology–based education are significantly effective in acquiring knowledge compared to traditional education groups, and also report significant effects on enhancing nursing students' confidence and self-efficacy. Although there was high heterogeneity among the studies for confidence and self-efficacy, the findings indicate the potential and feasibility of immersive technology–based education to improve learning outcomes in various aspects compared to traditional teaching methods, such as lectures and demonstrations.

### Comparison With Prior Work

With the growing prevalence of immersive technology, scholars have extensively assessed its effectiveness through systematic reviews and meta-analyses, particularly focusing on VR in educational settings [64-67]. However, prior to this study, comprehensive literature reviews considering immersive technologies beyond VR were scarce, with only 1 study addressing the learning outcomes associated with immersive technologies [2]. Therefore, it is essential to understand the educational components and effects of immersive technologies, including VR, AR, MR, and XR, given their expanding role in instructional practices.

Prior studies have often overlooked the categorization of VR based on immersion levels, despite the distinction between immersive, semi-immersive, and nonimmersive VR proposed by Cipresso et al [5]. Only a few studies have conducted literature reviews that consider the distinction of VR based on immersion levels [12,65]. Given the variability in educational effects based on the degree of immersion [68], it is crucial to classify VR according to the level of immersion to ascertain its educational impact. Therefore, this study specifically focused on VR, which uses HMDs, and investigated holistic educational effects without restrictions on scenarios or outcomes, contributing to a comprehensive understanding of the influence of immersive technology.

The results of interventions or research can be validated through the application of a theoretical framework, and it is more useful to determine distinctions under the same standard. Previous studies on nursing education, including several review studies, have assessed program effectiveness based on the NWKM [48,69-72]. Corresponding to the results of our study, the outcomes pertinent to levels 1 and 2 have been most frequently evaluated. Although the NWKM emphasizes the importance of level 4, few studies have explored level 4 outcomes [71,72]. Two studies conducted meta-analysis based on the NWKM: Delisle et al [62] compared the learning effectiveness of observers with active participants in health care simulation, finding no statistically significant differences in the subgroup analysis of NWKM level 2 outcomes. Piot et al [70] compared the learning effectiveness of various simulation types and reported that simulation effects on skills and attitudes, categorized as level 2, are more effective than comparisons, while the impact on knowledge does not reach statistical significance. However, this meta-analysis showed a significant effect on knowledge acquisition with immersive technology–based education compared to traditional teaching approach in RCTs. This finding suggests that among various types of simulations, particularly those using immersive technologies would significantly enhance learners' knowledge acquisition. Consequently, educators may find this evidence useful for integrating immersive technologies into their educational curricula. To the best of our knowledge, this study is the first to compare the effectiveness of traditional and immersive technologies.

### Conclusion

The results of this meta-analysis offer insights into the current application of immersive technology in nursing education among college nursing students. Additionally, the results show that immersive technology can contribute to improving knowledge acquisition, confidence, and self-efficacy. Notably, the outcome variables predominantly aligned with levels 1- 3 of the NWKM. Nevertheless, given the moderate-to-high heterogeneity among the studies included in the meta-analysis and the varying levels of evidence according to GRADE for each outcome, it is recommended that future well-designed RCTs be conducted and subsequent research be carried out.

## Appendix

Multimedia Appendix 1 Search term.

Multimedia Appendix 2 Intervention characteristics of included studies.

Multimedia Appendix 3 Preferred Reporting Items for Systematic Review and Meta-Analysis (PRISMA) checklist.

## Abbreviations

AI: artificial intelligence
AR: augmented reality
GRADE: Grading of Recommendations, Assessment, Development, and Evaluation
HMD: head-mounted device
MR: mixed reality
NWKM: New World Kirkpatrick Model
PRISMA: Preferred Reporting Items for Systematic Reviews and Meta-Analysis
RCT: randomized controlled trial
RoB 2: Risk-of-Bias Tool version 2
RoBANS: Risk-of-Bias Assessment Tool for Nonrandomized Studies
SMD: standardized mean difference
VR: virtual reality
XR: extended reality
