# Supplementary material for: Effects of Immersive Technology–Based Education for Undergraduate Nursing Students: Systematic Review and Meta-Analysis Using the Grading of Recommendations, Assessment, Development, and Evaluation (GRADE) Approach
Source: J Med Internet Res. 2024 Jul 24;26:e57566. doi: 10.2196/57566 (PMC11306947; doi:10.2196/57566)
Supplement: Multimedia Appendix 1 [file jmir_v26i1e57566_app1.docx]

| Multimedia Appendix 1. Summary of Database (DB) Search Terms. | |
| --- | --- |
| DB | Keywords |
| PubMed | ("students, nursing"[MeSH Terms] OR "nursing student*"[Title/Abstract] OR "student nurs*"[Title/Abstract] OR "undergraduate nurs*"[Title/Abstract]) AND ("virtual reality"[MeSH Terms] OR "augmented reality"[MeSH Terms] OR "virtual reality"[Title/Abstract] OR "augmented reality"[Title/Abstract] OR "virtual*"[Title/Abstract] OR "VR"[Title/Abstract] OR "AR"[Title/Abstract] OR "extended reality"[Title/Abstract] OR "mixed reality"[Title/Abstract] OR "immersive technology"[Title/Abstract] OR "metaverse"[Title/Abstract]) |
| Embase | ('virtual reality'/exp OR 'augmented reality'/exp OR 'virtual*':ab,ti OR 'vr':ab,ti OR 'augmented reality':ab,ti OR 'ar':ab,ti OR 'mixed reality':ab,ti OR 'extended reality':ab,ti OR 'immersive technology':ab,ti OR 'metaverse':ab,ti) AND ('nursing student'/exp OR 'nursing student' OR 'nursing student*':ab,ti OR 'student nurs*':ab,ti OR 'undergraduate nurs*':ab,ti) |
| CINAHL | (MH students, nursing OR TI nursing student* OR TI student nurs* OR TI undergraduate nur* OR AB nursing student* OR AB student nurs* OR AB undergraduate nur*) AND (MH virtual reality OR MH augmented reality OR TI virtual* OR TI "VR" OR TI augmented reality OR TI "AR" OR TI mixed reality OR TI extended reality OR TI immersive technology OR TI metaverse OR AB virtual* OR AB "VR" OR AB augmented reality OR AB "AR" OR AB mixed reality OR AB extended reality OR AB immersive technology OR AB metaverse) |
| Web of Science | ((TI=(nursing student* OR student nurs* OR undergraduate nurs*)) OR AB=(nursing student* OR student nurs* OR undergraduate nurs*)) AND ((TI=(virtual* OR "VR" OR augmented reality OR "AR" OR mixed reality OR extended realtiy OR immersive technology OR metaverse)) OR AB=(virtual* OR "VR" OR augmented reality OR "AR" OR mixed reality OR extended realtiy OR immersive technology OR metaverse)) |
