# Supplementary material for: Effects of Immersive Technology–Based Education for Undergraduate Nursing Students: Systematic Review and Meta-Analysis Using the Grading of Recommendations, Assessment, Development, and Evaluation (GRADE) Approach
Source: J Med Internet Res. 2024 Jul 24;26:e57566. doi: 10.2196/57566 (PMC11306947; doi:10.2196/57566)
Supplement: Multimedia Appendix 2 [file jmir_v26i1e57566_app2.docx]

**Table S1.** Intervention characteristics of included studies (N=23).

| Study | Intervention | Equipment | Comparison | Scenario (setting) | Simulation learning method (group/individual) | Length (minutes) | Featuring the entire patient’s view in the intervention section^a^ | Instructor | Prebriefing/debriefing^b^ | Outcomes^c^ |
| --- | --- | --- | --- | --- | --- | --- | --- | --- | --- | --- |
| Shujuan et al [39] | Disaster nursing VR^d^ training program: earthquake and fire, triage, wound dressing, fixation, hemostasis, debridement, cardiopulmonary resuscitation (CPR), tracheal intubation, transportation, decontamination, and supportive psychological care | HMD^e^ (HTC VIVE) | Traditional education and low-fidelity simulation | 2 disaster scenes and disaster nursing skills (disaster scenes of earthquake or fire) | Individual | 10-25 | O | NR^f^ | O/X | Knowledge (2, +), confidence (2, +), performance (2, +) |
| Havola et al [40] | VR simulation (VRS): assessing patient suffering from chest pain | HMD (VR headset) | None | Resuscitation (in addition to chest pain patient, intensive care unit [ICU]) | Individual | 16 (mean) | O | NR | O/X | Clinical reasoning (3, +) |
| Berg and Steinsbekk [41] | VirSam airway, breathing, circulation, disability, and exposure (ABCDE) application | HMD, hand controller | Self-learning with equipment | No scenario or setting specified | Individual | 20 | O | NR | O/X | Knowledge (2, –), satisfaction (1, NR), usability (1, +) |
| Berg and Steinsbekk [42] | VirSam ABCDE application | HMD, hand controller | Self-learning with equipment | No scenario or setting specified | Group | 20 | O | NR | O/X | Knowledge (2, –), satisfaction (1, NR), usability (1, +) |
| Ahn and Lee [43] | Home visit VR simulation (HVRS) program: community nursing curriculum application, core skills, health education, etc | Personal computer (PC), HMD, hand controller | Traditional education | Visiting nurse (patient’s home) | Individual | 8-20 | O | NR | O/O | Knowledge (2, +), performance (2, +), confidence (2, +), self-efficacy (2, +) |
| Jung and Park [44] | HMD-based VR nursing education program (VRP): see chemoport insertion surgery process in 360° | HMD | Self-learning | Chemoport insertion surgery nursing (angiography room) | Individual | NR | O | Researcher | O/O | Knowledge (2, +), attitude (2, +), satisfaction (1, +), motivation (2, –) |
| Lee and Han [45] | Mechanical ventilation VR nursing program: scenario 1 (high-pressure alarm), 2 (low-exhaled-volume alarm), 3 (high-respiration-rate alarm) | HMD (Oculus goggles) | Traditional education (video lecture) | Troubleshooting for ventilator alarms (ICU) | Individual | 15 | O | Researcher | O/O | Knowledge (2, –), self-efficacy (2, +), performance (2, +), satisfaction (1, +) |
| Yang and Oh [46] | VR neonatal resuscitation gamification program: Apgar score, aspiration, saturation monitoring, L-tube insertion, chest compression, intubation, medication | HMD (Oculus Rift S), hand controller | Traditional education | Neonatal resuscitation (neonatal care unit [NICU]) | Individual | 50 | O | NR | O/O | Knowledge (2, +), performance (2, –), clinical reasoning (3, +), confidence (2, +), motivation (2, +), anxiety (1, +) |
| Yu et al [47] | High-risk neonatal infection control (HirNIC) VRS | HMD (VIVE Pro Full-Kit, HTC VIVE), hand controller | Traditional education (routine NICU practice) | Basic nursing situations related to infection control (NICU) | Individual | 40 | O | Teacher | O/O | Knowledge (2, –), self-efficacy (2, +), satisfaction (1, +) |
| Yu and Yang [48] | VR infection control simulation (VRICS) program: donning and doffing personal protective equipment (PPE), respiratory care (patient’s status check and nasal-oral suction care) | HMD, hand controller | No intervention | Infection control; donning and doffing PPE; providing respiratory care for pediatric patients with COVID-19 (isolation unit with negative pressure system) | Individual | 90-110 | O | NR | O/O | Knowledge (2+), performance (2, +), self-efficacy (2, +), presence (1, –), satisfaction (1, –) |
| Rodríguez-Abad et al [49] | AR^g^ in learning about leg ulcer care: knowledge and skills | Smartphones or tablet PCs | Traditional education | No scenario or setting specified | Individual | 180 | X | NR | X/O | Performance (2, +), motivation (2, +) |
| Mayor Silva et al [50] | VRS for development of communications skills | HMD (VR goggles) | Traditional workshop | Communication skills performance (setting not specified) | Individual | NR | X | NR | O/O | Knowledge (2, +), performance (2, +) |
| Chang et al [51] | Spherical video-based VR (SVVR)–based childbirth learning system: childbirth education | HMD (VR glasses), mobile device | Traditional video approach | Childbirth (delivery room) | Individual | NR | X | NR | X/X | Knowledge (2, +), motivation (2, +), attitude (2, –), critical thinking (2, –), satisfaction (1, +) |
| Chao et al [52] | Immersive 3D interactive video program via VIVEPAPER: observe nasogastric (NG) tube feeding; program includes quizzes and information about physiology and anatomy relating to NG tube feeding | HMD (HTC VIVE) | Traditional education (watching NG tube feeding video) | NG tube feeding (clinical ward) | Individual | 10-20 | X | Researcher | O/X | Knowledge (2, –), confidence (2, –), satisfaction (1, +) |
| Chen et al [53] | 3D holograms | HMD (Microsoft Windows MR^h^ helmet), hand controller | Traditional education | Health assessment and practice (setting not specified) | Class lecture | NR | O | College lecturer | O/X | Knowledge (2, +), performance (2, +) |
| Chen and Liou [54] | AR pediatric first-aid training and evaluation system (AR-PFAES): includes Apgar score, aspiration, saturation monitoring, L-tube insertion, chest compression, intubation, medication | Mannequin, screen | Mannequin | First aid for choking and CPR (setting not specified) | Individual | 30 | O | NR | O/X | Knowledge (2, +), performance (2, +), confidence (2, +) |
| Wu et al [55] | VRS: administer oxygen, ensure safety (falls), vital signs, mental health, family education, wash hands, nursing record | Computers, HMD (Oculus Rift S) | Traditional education | Pediatric seizure management (pediatric ward) | Individual | 10-15 | O | NR | O/O | Knowledge (2, +) |
| Kurt and Öztürk [56] | Mobile AR: subcutaneous (SC), intramuscular (IM), and intravenous (IV) injections | Smartphone | Traditional education | No scenario or setting specified | Individual | NR | X | NR | X/O | Knowledge (2, +), performance (2, +) |
| Sen et al [57] | Mobile VR education program: surgical aseptic skills (washing hands, wearing sterile gloves, opening sterile packages, wearing the surgical gown, etc) | HMD (VR glasses), smartphones | Traditional education | Surgical aseptic skills (operating room) | Individual | 30 | X | NR | X/X | Knowledge (2, +), performance (2, +), confidence (2, +) |
| Dang et al [58] | Multisite evaluation of observer roles | HMD (VR goggles) | Traditional education (active participants/observer/audiovisual observer) | Patient complaining of lower leg pain after a surgical procedure (setting not specified) | Individual | 15 | X | NR | O/O | Knowledge (2, –), presence (1, +), usability (1, NR) |
| Herbert et al [59] | AR app on heart failure: anatomy and physiology of the heart | Smartphone | Traditional education (video lecture) | No scenario or setting specified | Individual | NR | X | NR | O/X | Knowledge (2, –), satisfaction (1, –) |
| Smith et al [60] | Disaster nursing skill of decontamination: donning PPE and performing actual decontamination | PC, HMD (Oculus Rift Developer Kit 2), hand controller | Traditional education | Decontamination in a disaster (emergency room) | Individual | 10 | O | NR | O/O | Knowledge (2, –), performance (2, –), satisfaction (1, NR) |
| Smith et al [61] | VRS in disaster training: donning PPE, cutting and removing decontaminated clothing, washing different areas of the patient in the proper order, and removing contaminated PPE in the proper order | HMD (Oculus Rift CV1), hand controller | Low-fidelity simulation (static mannequin) | Decontamination in disaster (setting not specified) | Individual | 10 | O | NR | O/X | Performance (2, –), satisfaction (1, –), self-efficacy (2, –) |

^a^O: the virtual patient’s entire view is visible; X: only part or none of the patient’s view is shown.

^b^O: prebriefing or debriefing was conducted; X: prebriefing or debriefing was not conducted.

^c^Outcomes: effectiveness of education program compared to comparison group; variables classified according to Kirkpatrick model levels, with “+” indicating *P*<.05 and “–” indicating *P*>.05.

^d^VR: virtual reality.

^e^HMD: head-mounted device.

^f^NR: not reported.

^g^AR: augmented reality.

^h^MR: mixed reality.
